# US Population Eligibility and Estimated Impact of Semaglutide Treatment on Obesity Prevalence and Cardiovascular Disease Events

**DOI:** 10.1007/s10557-023-07488-3

**Published:** 2023-08-14

**Authors:** Nathan D. Wong, Hridhay Karthikeyan, Wenjun Fan

**Affiliations:** Heart Disease Prevention Program, Division of Cardiology, C240 Medical Sciences, University of California, Irvine, CA 92697 USA

**Keywords:** Obesity, Cardiovascular disease, Prevention, Semaglutide, GLP-1 receptor agonists

## Abstract

**Background:**

Semaglutide 2.4 mg benefits weight loss and reduction of cardiovascular disease (CVD) risk factors in adults with obesity. We estimated the US population eligibility for semaglutide 2.4 mg (based on the weight management indication) and the impact on obesity and CVD events.

**Methods:**

We applied STEP 1 trial eligibility criteria to US adults aged ≥ 18 years in the US National Health and Nutrition Examination Survey (NHANES) 2015-2018 to estimate the US eligible population. Semaglutide weight changes in STEP 1 were applied to estimate the population impact on weight changes and obesity prevalence. We also estimated 10-year CVD risks utilizing the BMI-based Framingham CVD risk scores. The difference in estimated risks with and without semaglutide “treatment” multiplied by the eligible NHANES weighted population represented the estimated “preventable” CVD events.

**Results:**

We identified 3999 US adults weighted to an estimated population size of 93.0 million [M] (38% of US adults) who fit STEP 1 eligibility criteria. Applying STEP 1 treatment effects on weight loss resulted in an estimated 69.1% (64.3 M) and 50.5% (47.0 M) showing ≥ 10% and ≥ 15% weight reductions, respectively, translating to a 46.1% (43.0 M) reduction in obesity (BMI ≥ 30 kg/m^2^) prevalence. Among those without CVD, estimated 10-year CVD risks were 10.15% “before” and 8.34% “after” semaglutide “treatment” reflecting a 1.81% absolute (and 17.8% relative) risk reduction translating to 1.50 million preventable CVD events over 10 years.

**Conclusion:**

Semaglutide treatment in eligible US adults may substantially reduce obesity prevalence and CVD events, which may dramatically impact associated healthcare costs.

## Introduction

The prevalence of obesity in the United States adults in 2017–2020 is recently reported to be 41.9%, with the proportion of overweight or obese at 73.6% [1]. There are significant disparities by racial and ethnic groups, with obesity being highest in non-Hispanic black females (56.8%) [2]. The dramatic increase in obesity in the past 50 years is expected to continue. By 2030, every 1 in 2 US adults is expected to have obesity, with nearly 1 in 4 having severe obesity [3]. Moreover, the effects of obesity on cardiovascular disease (CVD) are well-established [4, 5], and it is important to further demonstrate the importance of body weight loss interventions on the prevention of CVD.

The evidence certain glucagon-like peptide 1 (GLP1) receptor agonists have in reducing cardiovascular outcomes in persons with diabetes [6, 7] has been of great interest. Recently, however, GLP1 receptor agonists [8, 9] in higher dosages than those used for diabetes that dramatically impact body weight loss have generated further interest and investigation. Semaglutide activates GLP1 receptors, improving incretin function and insulin secretion (glucose-dependent), inhibiting glucagon release and suppressing hepatic gluconeogenesis, resulting in reductions in both fasting as well as postprandial glucose [10]. The STEP 1 trial recently demonstrated the efficacy of once weekly semaglutide 2.4 mg in adults who are overweight or with obesity, showing a 14.9% reduction in body weight in the treated group compared to 2.4% in the placebo group [9]. There were also improvements in blood pressure, fasting plasma glucose, and lipids in the treated group relative to the placebo.

It is important to understand the eligible US population that could benefit from such therapy, its potential impact on the US population-wide prevalence of overweight/obesity, as well as the potential benefits on CVD outcomes. The purpose of the present study is to 1) identify the eligible US population based on the STEP 1 trial eligibility criteria and 2) project the population-wide impact on weight loss, obesity prevalence, and 3) estimate the preventable CVD events based on the cardiovascular risk factor effects seen in the Step 1 trial. Such information could further inform the implications of the ongoing SELECT cardiovascular outcomes trial [11].

## Methods

**Fig. 1:**
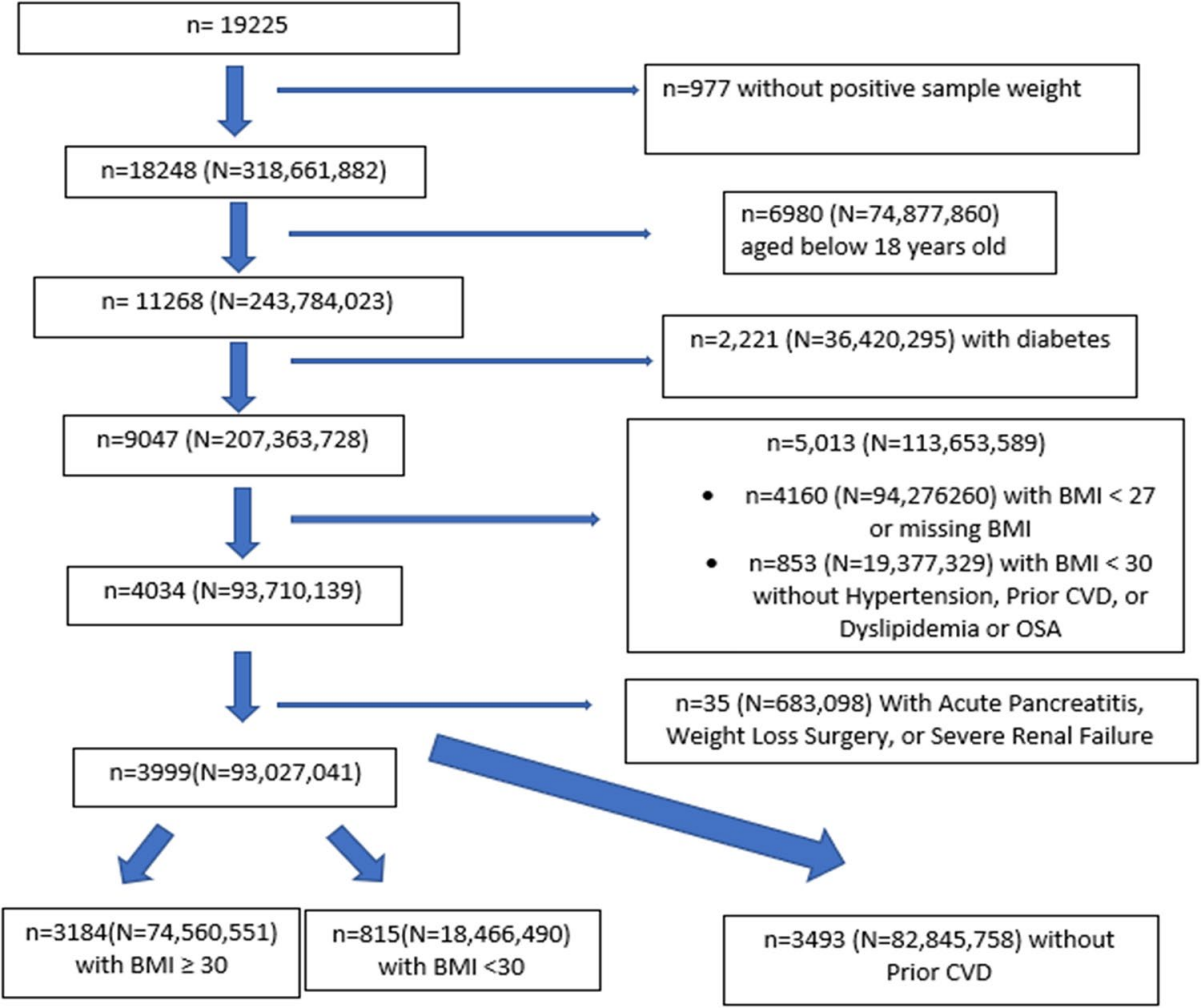
STEP 1 Eligible Sample Selection from the National Health and Nutrition Examination Surveys 2015–2018

We utilized data from the US National Health and Nutrition Examination Surveys (NHANES) 2015–2018, which are publicly available, and all participants gave prior consent to participate in NHANES*.* Inclusion criteria from the semaglutide STEP 1 trial [9] were applied to obtain our analytic sample (Fig. 1). Participants were adults aged 18 years of age or older with either a body mass index (BMI) of ≥ 30 kg/m^2^ or a BMI of ≥ 27 kg/m^2^ with at least one or more weight-related comorbidities of the following: known CVD, hypertension, dyslipidemia, or obstructive sleep apnea (as defined below). Patients with diabetes mellitus (DM), acute pancreatitis, bariatric surgery, or severe renal failure were excluded.

We estimated the number (with NHANES sample weighting applied to estimating the population in millions) and proportion of individuals in our projected US population sample who would be eligible for semaglutide based on the STEP 1 trial criteria and who are in the overweight (BMI ≥ 27 kg/m^2^ with known risks as described above) or with obesity (BMI of ≥ 30 kg/m^2^) categories, and by sex and ethnicity. NHANES provides data to define hypertension as blood pressure (BP) ≥ 130/80 mmHg or on medication; dyslipidemia as total cholesterol ≥ 200 mg/dL or triglycerides ≥ 200 mg/dL, or on lipid medication; CVD as a self-reported history of coronary heart disease, heart attack, stroke, or heart failure; and obstructive sleep apnea defined as occasional or frequent snore, snort or stopping breathing while asleep at least 3 times a week. Exclusion criteria available in NHANES were the following 1) history of DM based on an HbA1c of 6.5% or greater, fasting glucose ≥ 126 mg/dL, non-fasting glucose ≥ 200 mg/dL, or doctor told have DM, or taking DM medication or insulin, 2) history of pancreatitis or surgical obesity treatment, or 3) end stage kidney disease based on an eGFR < 15 ml/min.

We initially estimated how many US adults, in millions, would achieve weight reductions of ≥ 5%, ≥ 10%, and ≥ 15%, stratified by gender and race based on the proportions achieving these weight reductions in the semaglutide group in the STEP 1 trial [9]. We also determined estimated changes in obesity and overweight prevalence among our study sample after applying weight reductions from the STEP 1 trial semaglutide group to our study sample and comparing it to the obesity and overweight prevalences without “treatment”. Sex and ethnic-specific weight changes in STEP 1 have also been previously reported and were derived from a supplementary document [12].

Finally, based on the baseline level of CVD risk factors from our observed NHANES population applied to the Framingham risk scores for total CVD, we estimated in our NHANES sample without prior CVD the 10-year CVD event risk from the BMI-based Cox regression models published by D’Agostino and colleagues [13] (Appendix). This was repeated after applying the risk factor changes (in percent) from the STEP 1 trial [9] to estimate the “post-treatment” 10-year CVD risk. These risk factor changes (calculated from absolute body mass index and systolic blood pressure reductions in the semaglutide group) included -14.66% for BMI, and -4.89% for systolic blood pressure (SBP) for risk calculations based on BMI. This risk was multiplied by our eligible population to estimate the number of CVD events without semaglutide (baseline level) and with semaglutide (post-treatment), the difference being the preventable CVD events that would be expected from semaglutide treatment. These analyses were stratified by gender and race, based on the calculated BMI changes derived from the sex and ethnic-specific weight changes obtained as described above. Our primary analyses involved the use of the BMI-based models (given that weight reduction was the primary endpoint in the STEP 1 trial), and a sensitivity analysis was done using the laboratory-based Eqs. (13) (Appendix) and the respective Step 1 trial risk factor changes for total and high density lipoprotein (HDL)-cholesterol (of -3.0% and + 5.0%, respectively) instead of BMI. SAS version 9.4 was used to for our analyses. This study utilized publicly available de-identified data and was exempt from institutional review board review.

## Results

Among 19,225 participants in the NHANES 2015–18 we identified 3,999 (projected to 93.0 M) who fit STEP 1 trial eligibility criteria and were included in our analytic sample. Descriptive statistics on demographic and risk factor characteristics were obtained from the NHANES database and were presented for comparison along placebo group data from the STEP 1 trial (Table 1). While mean age was comparable between the STEP 1 trial and our NHANES sample (47 years), our NHANES sample had a smaller proportion who were female and of white or Asian race/ethnicity, while more were Black or Hispanic/Latino. Our NHANES sample also had higher proportion with pre-diabetes, but a lower mean BMI and lower proportions of participants with class II and class III obesity, as well as lower diastolic blood pressure (DBP), low-density lipoprotein cholesterol (LDL-C), and triglyceride levels compared to the STEP 1 group. Our NHANES sample tended to have fewer comorbidities than the STEP 1 sample.

**Table 1:** Descriptive Statistics among Step 1 Trial and NHANES Sample Participants

Characteristic	STEP 1 Trial Placebo* (N = 655)	NHANES Sample (N = 3999) 93.0 M
Age – Yr	47 ± 12	47.3 ± 0.5
Female Sex – No. (%)	498 (76.0)	2167 (50.6, 47.1 M)
Race or ethnic group — no. (%)		
White	499 (76.2)	1400 (63.3, 58.9 M)
Asian	80 (12.2)	231 (2.55, 2.38 M)
Black or African American	39 (6.0)	971 (12.0, 11.1 M)
Other	37 (5.6)	202 (4.93, 4.58 M)
Hispanic or Latino ethnic group — no. (%)	86 (13.1)	1195 (17.2, 16.1 M)
Body weight — kg	105.2 ± 21.5	97.8 ± 0.5
Body-mass index		
Mean	38.0 ± 6.5	34.4 ± 0.2
Distribution — no. (%)		
< 30	36 (5.5)	815 (19.9, 18.5 M)
≥ 30 to < 35 (Class I obesity)	207 (31.6)	1757 (44.6, 41.5 M)
≥ 35 to < 40 (Class II obesity)	208 (31.8)	818 (20.6, 19.2 M)
≥ 40 (Class III obesity)	204 (31.1)	609 (14.5, 13.9 M)
Waist circumference — cm	114.8 ± 14.4	111.4 ± 0.4
Glycated hemoglobin — %	5.7 ± 0.3	5.5 ± 0.008
Prediabetes — no. (%)	263 (40.2)	2002 (47.4, 44.1 M)
Blood pressure — mm Hg		
Systolic	127 ± 14	125.5 ± 0.4
Diastolic	80 ± 10	73.1 ± 0.4
Pulse — beats/min	72 ± 10	72.9 ± 0.3
Lipid levels		
Total cholesterol (mg/dL)	192.1 (19.4)	195.1 ± 1.5
HDL cholesterol (mg/dL)	49.5 (25.0)	50.8 ± 0.4
LDL cholesterol (mg/dL)	112.5 (29.8)	117.5 ± 0.9
Triglycerides (mg/dL)	127.9 (49.0)	119.7 ± 2.3
Estimated glomerular filtration rate ml/min/1.73 m^2^	95.9 (18.3)	95.0 ± 0.7
Coexisting conditions at the time of screening**		
Dyslipidemia — no. (%)	226 (34.5)	1561 (39.2, 36.5 M)
Hypertension — no. (%)	234 (35.7)	1688 (39.3, 36.6 M)
Knee osteoarthritis — no. (%)	102 (15.6)	491 (14.8, 13.8 M)
Obstructive sleep apnea — no. (%)	71 (10.8)	278 (6.7, 6.2 M)
Asthma or chronic obstructive pulmonary disease — no. (%)	80 (12.2)	495 (12.4, 11.6 M)
Nonalcoholic fatty liver disease — no. (%)	62 (9.5)	46 (1.2, 1.1 M)
Coronary artery disease — no. (%)	17 (2.6)	143 (3.1, 2.8 M)
No. of coexisting conditions at screening – no. (%)		
None	163 (24.9)	1292 (32.3, 30.0 M)
1	187 (28.5)	1340 (34.8, 32.4 M)
2	135 (20.6)	881 (20.5, 19.1 M)
3	96 (14.7)	367 (9.5, 8.8 M)
4	43 (6.6)	101 (2.4, 2.2 M)
≥ 5	31 (4.7)	18 (0.6, 0.54 M)

Continuous variables in the STEP 1 trial are reported as mean ± coefficient of variation and in NHANES as mean ± standard error. *STEP 1 trial placebo characteristics (which are similar to the intervention group characteristics in the trial) are shown for comparison with the NHANES eligible sample. For the STEP 1 sample, the Hispanic/Latino category presented is not mutually exclusive of the other race/ethnic groups. Categorical variables are reported as unweighted frequency (weighted percentage, weighted frequency in millions)

### Changes in Body Weight

**Fig. 2:**
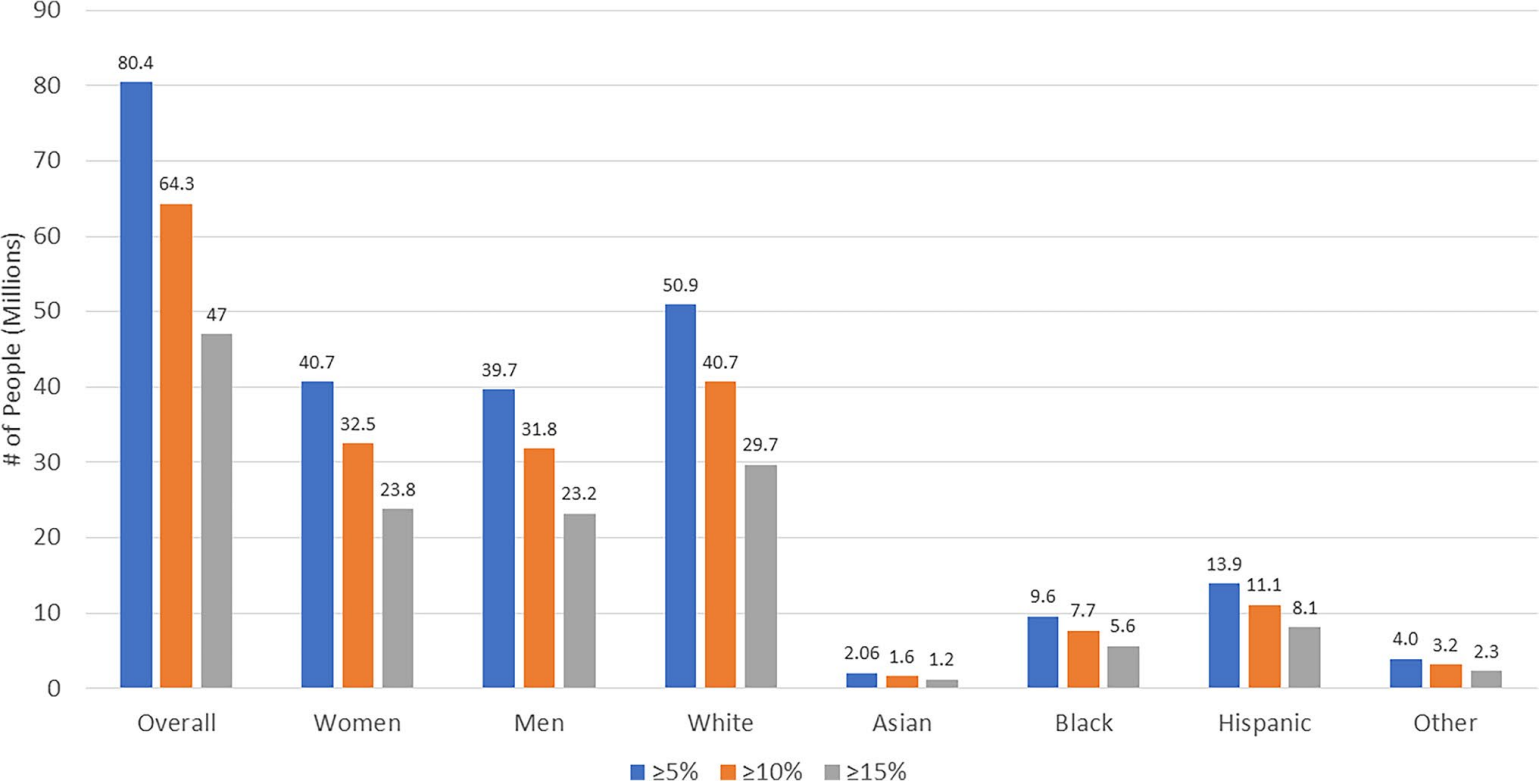
Number of US Adults (Millions) Estimated to have ≥ 5, ≥ 10, and ≥ 15% Body Weight Reductions from Semaglutide 2.4 mg Projected from the National Health and Nutrition Examination Surveys 2015-2018

Table 2 and Fig. 1 estimated the number of US adults from our STEP 1 eligible sample that would be expected to have ≥ 5%, ≥ 10%, and ≥ 15% weight reductions from semaglutide 2.4 mg treatment. Based on the STEP 1 trial results demonstrating 86.4%, 69.1%, and 50.5% of semaglutide treated persons would have ≥ 5%, ≥ 10%, and ≥ 15% weight reductions, respectively, among our projected sample of 93.0 million persons, we could estimate this to translate to 80.4 M, 64.3 M, and 47.0 M persons, respectively. Even if accounting for placebo effects, 41.9 M persons would be expected to have ≥ 15% weight reductions, and 51.8 M would have ≥ 10% weight reductions. The number of participants with each degree of weight reduction was roughly equal between males and females, with the greatest proportion being among white persons (Fig. 2).

**Table 2:** Estimated US Adults with ≥ 5, ≥ 10, and ≥ 15% Weight Reductions Based on STEP 1 Trial Placebo, Semaglutide, and Treatment Group Differences

Overall Sample (3999, 93.0 M)	Placebo (N = 3999) 93.0 M	Semaglutide (N = 3999) 93.0 M	Semaglutide-Placebo Diff
≥ 5%	31.5%/1260/32.9 M	86.4%/3455/80.4 M	54.9%/2195/47.5 M
≥ 10%	12.0%/480/12.5 M	69.1%/2763/64.3 M	57.1%/2283/51.8 M
≥ 15%	4.9%/196/5.1 M	50.5%/2019/47.0 M	45.6%/1823/41.9 M
Women	Placebo (N = 2167) 47.1 M	Semaglutide (N = 2167) 47.1 M	Semaglutide-Placebo Diff
≥ 5%	31.5%/683/14.8 M	86.4%/1872/40.7 M	54.9%/1189/25.9 M
≥ 10%	12.0%/260/5.7 M	69.1%/1497/32.5 M	57.1%/1237/26.9 M
≥ 15%	4.9%/106/2.3 M	50.5%/1094/23.8 M	45.6%/988/21.5 M
Men	Placebo (N = 1832) 46.0 M	Semaglutide (N = 1832) 46.0 M	Semaglutide-Placebo Diff
≥ 5%	31.5%/577/14.5 M	86.4%/1583/39.7 M	54.9%/1006/25.2 M
≥ 10%	12.0%/220/5.5 M	69.1%/1266/31.8 M	57.1%/1046/26.3 M
≥ 15%	4.9%/90/2.3 M	50.5%/925/23.2 M	45.6%/835/21.0 M
White	Placebo (N = 1400) 58.9 M	Semaglutide (N = 1400) 58.9 M	Semaglutide-Placebo Diff
≥ 5%	31.5%/441/18.6 M	86.4%/1210/50.9 M	54.9%/769/32.3 M
≥ 10%	12.0%/168/7.1 M	69.1%/967/40.7 M	57.1%/799/33.6 M
≥ 15%	4.9%/69/2.9 M	50.5%/707/29.7 M	45.6%/638/26.8 M
Asian	Placebo (N = 231) 2.38 M	Semaglutide (N = 231) 2.38 M	Semaglutide-Placebo Diff
≥ 5%	31.5%/73/0.8 M	86.4%/200/2.1 M	54.9%/127/1.3 M
≥ 10%	12.0%/28/0.3 M	69.1%/160/1.6 M	57.1%/132/1.4 M
≥ 15%	4.9%/11/0.1 M	50.5%/117/1.2 M	45.6%/106/1.1 M
Black	Placebo (N = 971) 11.1 M	Semaglutide (N = 971) 11.1 M	Semaglutide-Placebo Diff
≥ 5%	31.5%/306/3.5 M	86.4%/839/9.6 M	54.9%/533/6.1 M
≥ 10%	12.0%/117/1.3 M	69.1%/671/7.7 M	57.1%/554/6.3 M
≥ 15%	4.9%/48/0.54 M	50.5%/490/5.6 M	45.6%/442/5.1 M
Hispanic	Placebo (N = 1195) 16.1 M	Semaglutide (N = 1195) 16.1 M	Semaglutide-Placebo Diff
≥ 5%	31.5%/376/5.1 M	86.4%/1032/13.9 M	54.9%/656/8.8 M
≥ 10%	12.0%/143/1.9 M	69.1%/826/11.1 M	57.1%/683/9.2 M
≥ 15%	4.9%/59/0.79 M	50.5%/603/8.1 M	45.6%/544/7.3 M
Other	Placebo (N = 202) 4.58 M	Semaglutide (N = 202) 4.58 M	Semaglutide-Placebo Diff
≥ 5%	31.5%/64/1.4 M	86.4%/175/4.0 M	54.9%/111/2.5 M
≥ 10%	12.0%/24/0.55 M	69.1%/140/3.2 M	57.1%/116/2.6 M
≥ 15%	4.9%/10/0.22 M	50.5%/102/2.3 M	45.6%/92/2.1 M

### Changes in Overweight and Obesity Prevalence

**Fig. 3:**
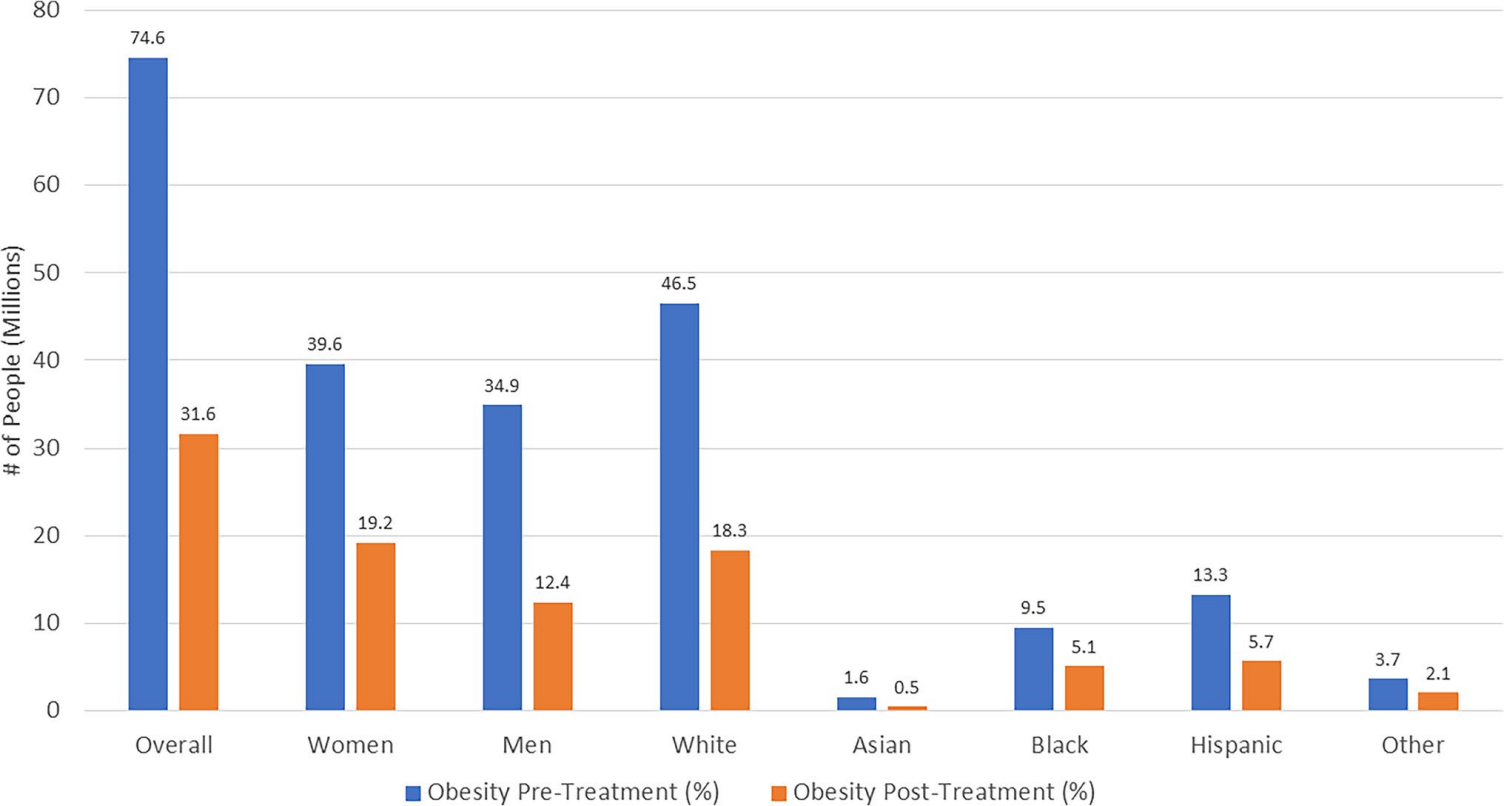
US Adults with Obesity, in Millions Before and After Semaglutide Treatment Based on Observed Weight Changes Projected from the National Health and Nutrition Examination Surveys 2015–2018

Among our study sample, “treatment” with semaglutide 2.4 mg weekly was estimated to be associated with substantial reductions in obesity prevalence of 46.1%, corresponding to 43.0 M fewer persons with obesity, with many of these transitioning to the overweight category (where there was an increased prevalence of 29.3%). Importantly, 16.8% of our sample, corresponding to 17.5 M persons would now be expected to be in the normal weight category (Table 3 and Fig. 3). Sex stratified analyses showed a roughly equivalent number of women (20.4 M) and men (22.5 M) no longer with obesity after semaglutide “treatment".

**Table 3:** Proportion and Number of US Adults (n and millions) Pre and Post-Semaglutide Treatment in Each Weight Category Based on Observed Weight Changes

Overall Sample: (3999, 93.0 M)	Pre-Treatment	Post-Treatment	Difference
Normal (< 25)	0%/0/0.00 M	16.8%/630/17.5 M	+ 16.8%/ + 630/ + 17.5 M
Overweight (25 to < 30)	19.9%/815/18.5 M	49.2%/1955/45.7 M	+ 29.3%/ + 1140/ + 27.2 M
Obese (≥ 30)	80.1%/3184/74.6 M	34.0%/1414/31.6 M	-46.1%/-1770/-43.0 M
Women: (2167, 47.1 M)	Pre-Treatment	Post-Treatment	Difference
Normal (< 25)	0%/0/0.0 M	13.1%/274/6.2 M	+ 13.1/ + 274/6.2 M
Overweight (25 to < 30)	15.8%/355/7.4 M	46.2%/1003/21.7 M	+ 30.4%/ + 648/ + 14.3 M
Obese (≥ 30)	84.2%/1812/39.6 M	40.8%/890/19.2 M	-43.4%/-922/-20.4 M
Men: (1832, 46.0 M)	Pre-Treatment	Post-Treatment	Difference
Normal (< 25)	0%/0/0.0 M	20.7%/356/9.52 M	+ 20.7%/ + 356/ + 9.5 M
Overweight (25 to < 30)	24.0%/460/11.0 M	52.3%/952/24.0 M	+ 28.3%/ + 492/ + 13.0 M
Obese (≥ 30)	76.0%/1372/34.9 M	27.0%/524/12.4 M	-49.0%/-848/-22.5 M
White: (1400, 58.9 M)	Pre-Treatment	Post-Treatment	Difference
Normal (< 25)	0%/0/0.0 M	19.7%/288/11.6 M	+ 19.7%/ + 288/ + 11.6 M
Overweight (25 to < 30)	21.1%/313/12.4 M	49.3%/669/29.0 M	+ 28.2%/ + 356/ + 16.6 M
Obese (≥ 30)	78.9%/1087/46.5 M	31.1%/443/18.3 M	-47.8%/-644/-28.2 M
Asian: (231, 2.38 M)	Pre-Treatment	Post-Treatment	Difference
Normal (< 25)	0%/0/0.0 M	14.7%/39/0.4 M	+ 14.7%/ + 39/ + 0.4 M
Overweight (25 to < 30)	32.9%/83/0.78 M	64.6%/146/1.5 M	+ 31.7%/ + 63/ + 0.8 M
Obese (≥ 30)	67.1%/148/1.6 M	20.7%/46/0.5 M	-46.4%/-102/-1.1 M
Black: (971, 11.1 M)	Pre-Treatment	Post-Treatment	Difference
Normal (< 25)	0%/0/0.0 M	11.3%/125/1.3 M	+ 11.3%/ + 125/ + 1.3 M
Overweight (25 to < 30)	14.4%/158/1.6 M	43.1%/427/4.8 M	+ 28.7%/ + 269/ + 3.2 M
Obese (≥ 30)	85.6%/813/9.5 M	45.6%/419/5.1 M	-40.0%/-394/-4.5 M
Hispanic: (1195, 16.1 M)	Pre-Treatment	Post-Treatment	Difference
Normal (< 25)	0%/0/0.0 M	12.5%/165/2.0 M	+ 12.5%/ + 165/ + 2.0 M
Overweight (25 to < 30)	17.3%/231/2.8 M	52.0%/628/8.4 M	+ 34.7%/ + 397/5.6 M
Obese (≥ 30)	82.7%/964/13.3 M	35.4%/402/5.7 M	-47.3%/-562/-7.6 M
Other: (202, 4.58 M)	Pre-Treatment	Post-Treatment	Difference
Normal (< 25)	0%/0/0.0 M	10.2%/13/0.47 M	+ 10.2%/ + 13/ + 0.47 M
Overweight (25 to < 30)	19.9%/30/0.91 M	44.8%/85/2.1 M	+ 24.9%/ + 55/ + 1.1 M
Obese (≥ 30)	80.1%/172/3.7 M	45.0%/104/2.1 M	-35.1%/-68/-1.6 M

### Changes in CVD 10-year Risk and Preventable CVD Events

Among our sample, 3,493 (82.8 M) were without prior CVD and eligible for CVD 10-year risk estimation as described above. 10-year CVD risk (%) estimates before and after “treatment” in our study sample are shown in Table 4 along with the corresponding numbers of estimated CVD events, with the difference being the “preventable” CVD events. The average risk for the whole sample pre-treatment was 10.15%, being expectedly lower in females and higher in males, with whites having the highest average risk, with the corresponding number of estimated CVD events in 10 years reflecting this. Application of the risk factor changes in STEP 1 yielded an overall risk reduction of 1.81% (and a relative risk reduction of 17.8%), with the greatest absolute reduction in risk among males (2.20%) and white persons (2.01%). With an estimated 8.41 M events without treatment with semaglutide, compared to 6.91 M events with treatment, we estimate a total number of preventable CVD events of 1.50 M, with most of these events being prevented among males (0.91 M) and among white persons (1.01 M). In a sensitivity analysis using the laboratory-based equations that utilize total and HDL-C cholesterol (and their respective changes as observed from semaglutide treatment in the STEP 1 trial) instead of BMI, estimated pre-treatment and post-treatment CVD risks were 7.62% and 6.52% for an absolute risk reduction of 1.10% (and a relative risk reduction of 14.4%), with an estimated preventable CVD events of 0.86 M. Absolute risk reduction was greater among males (1.28%) than females (0.92%), with absolute risk reductions of 1.28% and 0.92%, respectively. White persons also had the greatest absolute risk reduction (1.23%) and number of projected preventable CVD events (0.60 M).

**Table 4:** Estimated Cardiovascular Events and Preventable Events, based on BMI parameters

	*n* (M)	CVD Risk Pre-Treatment (%)	CVD Risk Post-Treatment (%)	Difference	CVD Events Pre	CVD Events Post	Difference
Overall	3493 (82.8M)	10.15%	8.34%	1.81%	355 (8.41 M)	291 (6.91 M)	63 (1.50 M)
Females	1915 (42.2M)	7.64%	6.24%	1.41%	146 (3.22 M)	119 (2.63 M)	27 (0.59 M)
Males	1578 (41.5M)	12.71%	10.51%	2.20%	201 (5.27 M)	166 (4.36 M)	35 (0.91 M)
Whites	1187 (50.1M)	11.60%	9.59%	2.01%	138 (5.81 M)	114 (4.80 M)	24 (1.01 M)
Asians	210 (2.1M)	7.07%	5.79%	1.28%	15 (0.15 M)	12 (0.12 M)	3 (0.03 M)
Blacks	833 (9.8M)	9.00%	7.39%	1.61%	75 (0.88 M)	62 (0.72 M)	13 (0.16 M)
Hispanic	1087 (14.8M)	6.95%	5.68%	1.27%	76 (1.03 M)	62 (0.84 M)	14 (0.19 M)
Other	176 (4.0M)	6.64%	5.43%	1.21%	12 (0.27 M)	10 (0.22 M)	2 (0.05 M)

Estimates combining strata may not total overall due to rounding error

## Discussion

Our study suggests that approximately 93 million US adults with overweight and obesity would be potentially eligible for semaglutide 2.4 mg for weight loss based on STEP 1 eligibility criteria. Approximately 80, 64, and 47 million of these persons would be expected to experience ≥ 5%, ≥ 10% or ≥ 15% weight reductions, respectively, and nearly half (46% representing 43 million persons) of those initially with obesity would no longer have obesity after treatment. Moreover, we estimate up to 1.5 million CV events could be prevented over a 10-year period from the expected risk factor changes resulting from such treatment, based on our estimate of a 1.8% absolute risk reduction (and corresponding 17.8% relative risk reduction). These findings have important implications for the potential US population-wide impact of semaglutide 2.4 mg therapy for persons with overweight or obesity.

There has been great interest in GLP1-RA and even more novel therapies to treat obesity. While once daily liraglutide 3.0 mg was initially approved for obesity, more recently, once weekly semaglutide 2.4 mg has been approved. In general, weight reduction from GLP1-RA therapy in those without diabetes (but with overweight or obesity) tends to be greater (6.1–7.4%) than in those with diabetes (4% to 6.2%) [14]. In the SCALE study involving 3.0 mg daily liraglutide for persons without DM, but with BMI ≥ 30 kg/m^2^ or ≥ 27 kg/m^2^ if with dyslipidemia or hypertension, those on liraglutide lost an average of 8% of their body weight, with 63%, 33%, and 14% losing at ≥ 5%, > 10%, and > 15% of body weight, respectively [15]. Using similar BMI cutpoints for semaglutide 2.4 mg in the STEP 1 trial, there was an overall 15.3 kg weight loss corresponding to a 14.9% reduction, with 88.4% and 69.1% showing weight reductions of at least 5% and 10%, respectively [9]. More recently, in the SURMOUNT-1 trial, the novel dual glucose-dependent insulinotropic polypeptide (GIP) and glucagon-like peptide-1 (GLP-1) receptor agonist (RA) tirzepatide showed a mean 15.0% reduction in weight with 91% of participants on the 15 mg once weekly dosage having at least 5% weight loss; 57% of participants had 20% or more body weight reductions [16].

GLP1-RA as well as GIP-GLP-1 RA therapies also show improvements in cardiometabolic risk factors which may explain their cardiovascular risk reduction potential. In the SCALE study, liraglutide 3.0 mg daily showed reductions of systolic and diastolic blood pressure of 4.2 and 2.6 mmHg, respectively, and reductions in LDL-cholesterol and triglycerides of 3.0% and 13.3%, respectively, and increases in HDL-cholesterol of 2.3% [15]. In STEP 1 involving semaglutide, there was a reduction in body mass index of 5.5 kg/m2, with corresponding reductions in systolic blood pressure of 6.2 mmHg along with reductions in total and LDL-cholesterol of 3% each, increases in HDL-cholesterol of 5%, and reductions in triglycerides of 22% [9]. In the SURMOUNT-1 trial [16], among the pooled tirzepatide groups, there was a 7.2 mmHg reduction in systolic blood pressure with reductions in non-HDL-C and triglycerides of 9.7% and 24.8%, respectively, and increases in HDL-C of 8.0%. The ongoing SELECT trial [11] will inform how semaglutide therapy and its associated risk factor changes translates into cardiovascular outcomes in adults with obesity and established CV disease and without diabetes. 

There are limited real-world data on the population-wide impact of these therapies, although some data exist on the use of these therapies specifically in persons with diabetes. In an analysis of the US Diabetes Collaborative Registry [17], Arnold and colleagues studied 182,525 patients with diabetes in the US Diabetes Collaborative Registry that included 313 cardiology, endocrinology, and primary care practices and noted that 48% of such patients fit LEADER eligibility criteria; use among such patients was estimated to potentially prevent 247 MIs and 329 CV deaths per year of treatment (or 300 MIs and 400 CV deaths per 100,000 eligible patients treated for 1 year). Moreover, Fan et al. [18], estimated liraglutide eligibility and potential preventable events among the US National Health and Nutrition examination estimated population of 27.3 million persons with diabetes, 15.4% (4.2 million) fit LEADER eligibility criteria, and based on LEADER cardiovascular outcome risk reductions from the 1.8 mg liraglutide (or maximally tolerated dose) used in the trial, we estimated 21,209 primary composite CVD events, 29,691 extended CVD composite outcomes, 16,967 all-cause deaths, 16,967 cardiovascular deaths, and 12,725 myocardial infarctions could potentially be prevented annually. Finally, in a recent analysis of NHANES, Lu et al. [19] showed 51.1% of US adults were estimated to meet the Food and Drug Administration eligibility criteria for semaglutide 2.4 mg, with the percentage highest among Black adults (56.6%), followed by Hispanic adults (55.0%), with overall 11.9% being uninsured, 13.3% lacking a usual source of care, 33.6% having low family income, and 38.9% lacking higher education. The current study provides further insight into the potential impact of semaglutide-eligible US adults on obesity prevalence and CVD outcomes.

This study has important strengths, limitations, and assumptions. A key strength of this study is the utilization of the NHANES cohort of US adults which provides sample weighting allowing US population estimation of STEP 1 eligible US adults and preventable CVD events among the ethnically diverse US population. While NHANES does utilize self-reported measures for certain factors, including CVD status, the reliability of such self-report measures has been confirmed previously [20]; however, our key measures that do impact on cardiovascular risk status, namely weight (for calculation of body mass index), blood pressure, and lipids, are all measured variables in NHANES. Since NHANES included only baseline assessments, the impact of changes in factors such as caloric intake cannot be assessed, nor was this a factor assessed in the STEP 1 trial. However, one limitation is that while we are utilizing STEP 1 eligibility criteria to identify the eligible NHANES participants, there may be differences between our participants and the STEP 1 clinical trial participants, and therefore the weight loss and cardiovascular risk factor changes observed in STEP 1 may not be entirely translatable to our NHANES sample. For instance, our sample had fewer comorbidities as well as a lower proportion of more severely obese individuals. This would be expected to result in our study sample being of lower CVD risk at baseline, along with lower absolute projected reductions in risk and estimated CVD events than that which would have been predicted had our participants more closely matched those enrolled in the STEP 1 trial. As we are applying STEP 1 trial treatment effects to our NHANES sample, we are assuming the treatment effects will be the same in our cohort as in the STEP 1 trial. In addition, since we are applying the semaglutide group treatment effects in our estimations of preventable CVD events, it is uncertain whether our estimated preventable CVD events can be assumed to be due exclusively to semaglutide, or could be the result of other risk factor interventions. We also used the actual semaglutide group treatment effects (rather than placebo-adjusted) which are felt to better represent the real-world effects we are projecting the STEP 1 trial results to. Finally, while the Framingham Risk Scores are a standard for estimating CVD events, like most risk scores, they are not as accurate as capturing actual CVD events as would be the case in a true cardiovascular outcomes clinical trial. The equations (as well as other risk scores) do not include all possible factors (e.g., family history or other “risk enhancing” factors) that could affect CVD risk. Advantages of using the Framingham total CVD risk score [12] to project CVD risk over other equations is its *inclusion of persons beginning age 20* and *prediction of total CVD events* (not just myocardial infarction and stroke, but also angina, heart failure and peripheral arterial disease are included in the outcome as they are also important CVD manifestations) instead of only hard ASCVD events as most other CVD risk scores feature. While our sensitivity analysis suggests lower risks and a lower number of preventable CVD events from using laboratory-based equations incorporating total and HDL-cholesterol changes instead of BMI changes, it is possible that treatment for hypercholesterolemia in a number of subjects (17% were estimated to be on cholesterol-lowering medication) may have resulted in lower cholesterol levels and underestimated risks. Moreover, estimating risks based on BMI changes can additionally reflect changes in risk due to on changes in triglycerides and inflammatory factors such as c-reactive protein, which are significantly impacted by semaglutide treatment.

In conclusion, our study suggests over 90 million US adults with overweight or obesity would be potentially eligible for semaglutide treatment for chronic weight management. Such treatment could reduce by nearly half the size of the population with obesity, as well as prevent up to 1.5 million CVD events if treated for 10 years. This could have a significant impact on reducing healthcare costs associated with obesity and CVD. The ongoing SELECT trial [10] will document the actual impact of semaglutide treatment on cardiovascular outcomes in high-risk adults and established CVD and without diabetes.

## Data Availability

Data used in this study are publicly available from the National Health and Nutrition Examination Survey: https://www.cdc.gov/nchs/nhanes/index.htm.

## Appendix

Equations Used for Obtaining CVD 10-Year Risk, Based on the Cox Model (based on D’Agostino RB et al. [13])

**BMI-Based Calculations:**

**Women:**

SBPBeta for Treatment = 2.88267; SBPBeta for Non-Treatment = 2.81291;

RiskPart = 2.72107*log(Age) + 0.51125*log(BMI) + SBPBeta*log(SBP) + 0.61868*Smoker + 0.77763*Diabetes.

CVDRisk = 1–0.94833.^exp^^(RiskPart−26.0145)^

**Men:**

SBPBeta for Treatment = 1.92672; SBPBeta for Non-Treatment = 1.85508;

RiskPart = 3.11296*log(Age) + 0.79277*log(BMI) + SBPBeta*log(SBP) + 0.70953*Smoker + 0.53160*Diabetes;

CVDRisk = 1–0.88431.^exp^^(RiskPart−23.9388)^

**Laboratory-Based (Cholesterol) Calculations:**

**Women:**

SBPBeta for Treatment = 2.82263; SBPBeta for Non-Treatment = 2.76157;

RiskPart = 2.32888*log(Age) + 1.20904*log(Total Cholesterol)-0.70833*log(HDL) + SBPBeta*log(SBP) + 0.61868*Smoker + 0.77763*Diabetes;

CVDRisk = 1–0.95012.^exp^^(RiskPart−26.1931)^

**Men:**

SBPBeta for Treatment = 1.99881; SBPBeta for Non-Treatment = 1.93303;

RiskPart = 3.06117*log(Age) + 1.12370*log(Total Cholesterol)-0.93263*log(HDL) + SBPBeta*log(SBP) + 0.70953*Smoker + 0.53160*Diabetes;

CVDRisk = 1–0.88936.^exp^^(RiskPartChol−23.9802)^

## Code Availability

Applicable code may be provided by the authors upon request.

## References

[1] NHANES Fast Stats Overweight and Obesity. Data derived from National Health and Nutrition Examination Survey 2017–March 2020 Prepandemic Data Files—Development of Files and Prevalence Estimates for Selected Health Outcomes, National Health Statistics Reports No. 158, June 14, 2021. https://www.cdc.gov/nchs/fastats/obesity-overweight.htm. Accessed 2/2/23. 10.15620/cdc:106273 PMC11513744 39380201

[2] Tsao CW, Aday AW, Almarzooq ZI, American Heart Association Council on Epidemiology and Prevention Statistics Committee and Stroke Statistics Subcommittee, et al. Heart Disease and Stroke Statistics-2023 Update: A Report From the American Heart Association. Circulation. 2023;147(8):e93–621. 36695182 10.1161/CIR.0000000000001123 PMC12135016

[3] Ward ZJ, Bleich SN, Cradock AL, et al. Projected U.S. state-level prevalence of adult obesity and severe obesity. N Engl J Med. 2019;381:2440–50. 31851800 10.1056/NEJMsa1909301

[4] Afshin A, Forouzanfar MH, Reitsma MB, et al. Health effects of overweight and obesity in 195 countries over 25 years. N Engl J Med. 2017;377(1):13–27. 28604169 10.1056/NEJMoa1614362 PMC5477817

[5] Jensen MD, Ryan DH, Apovian CM, et al. 2013 AHA/ACC/TOS guideline for the management of overweight and obesity in adults: a report of the American College of Cardiology/American Heart Association Task Force on Practice Guidelines and the Obesity Society. J Am Coll Cardiol. 2014;63(Pt B):2985–3023. 24239920 10.1016/j.jacc.2013.11.004

[6] Marso SP, Daniels GH, Brown-Frandsen K, et al. Liraglutide and cardiovascular outcomes in type 2 diabetes. N Engl J Med. 2016;375(4):311–22. 27295427 10.1056/NEJMoa1603827 PMC4985288

[7] Marso SP, Bain SC, Consoli A, SUSTAIN-6 Investigators, et al. Semaglutide and cardiovascular outcomes in patients with type 2 diabetes. N Engl J Med. 2016;375(19):1834–44. 27633186 10.1056/NEJMoa1607141

[8] Rye P, Modi R, Cawsey S, Sharma AM. Efficacy of high-dose liraglutide as an adjunct for weight loss in patients with prior bariatric surgery. Obes Surg. 2018;28(11):3553–8. 30022424 10.1007/s11695-018-3393-7

[9] Wilding JH, Batterham RL, Calanna S, et al. One-weekly semaglutide in adults with overweight or obesity. N Engl J Med. 2021;384:989–1002. 33567185 10.1056/NEJMoa2032183

[10] Knudsen LB, Lau J. The discovery and development of liraglutide and semaglutide. Front Endocrinol (Lausanne). 2019;10:155. 31031702 10.3389/fendo.2019.00155 PMC6474072

[11] Ryan DH, Lingvay I, Colhoun HM, et al. Semaglutide effects on cardiovascular outcomes in people with overweight or obesity (SELECT) rationale and design. Am Heart J. 2020;229:61–9. 32916609 10.1016/j.ahj.2020.07.008

[12] Kushner RF, Garvey WT, Hesse D, et al. Once-weekly Subcutaneous Semaglutide 2.4 mg Reduces Body Weight in Adults with Overweight or Obesity Regardless of Baseline Characteristics (STEP 1). Poster 7196 presented at: ENDO 2021; March 20-23, 2021 and Data on File, Novo Nordisk

[13] D’Agostino RB Sr, Vasan RS, Pencina MJ, Wolf PA, Cobain M, Massaro JM, Kannel WB. General cardiovascular risk profile for use in primary care: the Framingham Heart Study. Circulation. 2008;117(6):743–53. 18212285 10.1161/CIRCULATIONAHA.107.699579

[14] Jensterle M, Rizzo M, Haluzík M, Janež A. Efficacy of GLP-1 RA approved for weight management in patients with or without diabetes: a narrative review. Adv Ther. 2022;39(6):2452–67. 35503498 10.1007/s12325-022-02153-x PMC9063254

[15] Pi-Sunyer X, Astrup A, Fujioka K, Greenway F, Halpern A, Krempf M, Lau DC, le Roux CW, Violante Ortiz R, Jensen CB, Wilding JP, SCALE Obesity and Prediabetes NN8022-1839 Study Group. A randomized, controlled trial of 3.0 mg of liraglutide in weight management. N Engl J Med. 2015;373(1):11–22. 26132939 10.1056/NEJMoa1411892

[16] Jastreboff AM, Aronne LJ, Ahmad NN, Wharton S, Connery L, Alves B, Kiyosue A, Zhang S, Liu B, Bunck MC, SURMOUNT-1 Investigators. Stefanski a tirzepatide once weekly for the treatment of obesity. N Engl J Med. 2022;387(3):205–16. 35658024 10.1056/NEJMoa2206038

[17] Arnold SV, Inzucchi SE, Tang F, McGuire DK, Mehta SN, Maddox TM, Goyal A, Sperling LS, Einhorn D, Wong ND, Khunti K, Lam CS, Kosiborod M. Real-world use and modeled impact of glucose-lowering therapies evaluated in recent cardiovascular outcomes trials: An NCDR® Research to Practice project. Eur J Prev Cardiol. 2017;24(15):1637–45. 28870145 10.1177/2047487317729252

[18] Fan W, Tong C, Wong ND. LEADER trial eligibility and preventable cardiovascular events in US adults with diabetes: the national health and nutrition examination surveys 2007–2016. Cardiovasc Drugs Ther. 2020;34(6):737–43. 32621045 10.1007/s10557-020-07032-7

[19] Lu Y, Liu Y, Krumholz HM. Racial and ethnic disparities in financial barriers among overweight and obese adults eligible for semaglutide in the United States. J Am Heart Assoc. 2022;11(19):e025545. 36172953 10.1161/JAHA.121.025545 PMC9673703

[20] Bergmann MM, Byers T, Freedman DS, Mokdad A. Validity of self-reported diagnoses leading to hospitalization: a comparison of self-reports with hospital records in a prospective study of american adults. Am J Epidemiol. 1998;147(10):969–77. 9596475 10.1093/oxfordjournals.aje.a009387

